# GDF-15 Is Elevated in Children with Mitochondrial Diseases and Is Induced by Mitochondrial Dysfunction

**DOI:** 10.1371/journal.pone.0148709

**Published:** 2016-02-11

**Authors:** Raquel Montero, Delia Yubero, Joan Villarroya, Desiree Henares, Cristina Jou, Maria Angeles Rodríguez, Federico Ramos, Andrés Nascimento, Carlos Ignacio Ortez, Jaume Campistol, Belen Perez-Dueñas, Mar O'Callaghan, Mercedes Pineda, Angeles Garcia-Cazorla, Jaume Colomer Oferil, Julio Montoya, Eduardo Ruiz-Pesini, Sonia Emperador, Marija Meznaric, Laura Campderros, Susana G. Kalko, Francesc Villarroya, Rafael Artuch, Cecilia Jimenez-Mallebrera

**Affiliations:** 1 Clinical Biochemistry Department, Hospital Sant Joan de Déu, Barcelona, Spain; 2 Center for Biomedical Research on Rare Diseases (CIBERER), Madrid, Spain, Instituto de Salud Carlos III, Madrid, Spain; 3 Institute of Pediatric Research Sant Joan de Déu, Barcelona, Spain; 4 Biochemistry and Molecular Biology Department, Biomedical Institute University of Barcelona (IBUB), Center for Biomedical Research on Obesity and Nutrition (CIBEROBN), Madrid, Spain; 5 Neuromuscular Unit, Neuropaediatrics Department, Hospital Sant Joan de Déu, Fundacion Sant Joan de Deu, Barcelona, Spain; 6 Pathology Department, Hospital Sant Joan de Déu, Barcelona, Spain; 7 Neuropaediatrics Department, Hospital Sant Joan de Déu, Barcelona, Spain; 8 Biochemistry and Molecular Biology Department, University of Zaragoza, Zaragoza, Spain; 9 Fundación ARAID, Universidad de Zaragoza, Zaragoza, Spain; 10 Institute of Anatomy, Faculty of Medicine, University of Ljubljana, Ljubljana, Slovenia; 11 Bioinformatics Core Facility, IDIBAPS, Hospital Clinic, Barcelona, Spain; Institut de Recerca de la Santa Creu i Sant Pau, SPAIN

## Abstract

**Background:**

We previously described increased levels of growth and differentiation factor 15 (GDF-15) in skeletal muscle and serum of patients with mitochondrial diseases. Here we evaluated GDF-15 as a biomarker for mitochondrial diseases affecting children and compared it to fibroblast-growth factor 21 (FGF-21). To investigate the mechanism of GDF-15 induction in these pathologies we measured its expression and secretion in response to mitochondrial dysfunction.

**Methods:**

We analysed 59 serum samples from 48 children with mitochondrial disease, 19 samples from children with other neuromuscular diseases and 33 samples from aged-matched healthy children. GDF-15 and FGF-21 circulating levels were determined by ELISA.

**Results:**

Our results showed that in children with mitochondrial diseases GDF-15 levels were on average increased by 11-fold (mean 4046pg/ml, 1492 SEM) relative to healthy (350, 21) and myopathic (350, 32) controls. The area under the curve for the receiver-operating-characteristic curve for GDF-15 was 0.82 indicating that it has a good discriminatory power. The overall sensitivity and specificity of GDF-15 for a cut-off value of 550pg/mL was 67.8% (54.4%-79.4%) and 92.3% (81.5%-97.9%), respectively. We found that elevated levels of GDF-15 and or FGF-21 correctly identified a larger proportion of patients than elevated levels of GDF-15 or FGF-21 alone. GDF-15, as well as FGF-21, mRNA expression and protein secretion, were significantly induced after treatment of myotubes with oligomycin and that levels of expression of both factors significantly correlated.

**Conclusions:**

Our data indicate that GDF-15 is a valuable serum quantitative biomarker for the diagnosis of mitochondrial diseases in children and that measurement of both GDF-15 and FGF-21 improves the disease detection ability of either factor separately. Finally, we demonstrate for the first time that GDF-15 is produced by skeletal muscle cells in response to mitochondrial dysfunction and that its levels correlate *in vitro* with FGF-21 levels.

## Introduction

Mitochondrial diseases are a group of heterogeneous disorders in which the main feature is dysfunction of the mitochondrial respiratory chain leading to defective ATP production. They are the most common group of metabolic diseases with an estimated prevalence in the population of approximately 1 in 5000. Clinically, they are characterized by multisystemic involvement generally affecting tissues with high energy demand [1], [2].

Diagnosis of mitochondrial diseases is very complex. This is particularly true in children due to the complexity of the clinical presentations and lack of classical diagnostic clues such as ragged-red fibres in muscle biopsy. Accurate guidelines have been published which take into account clinical symptoms and biochemical and histopathological criteria [3], [4], [5]. Although the gold-standard is genetic confirmation often the genetic basis of the disease remains unidentified [6], [7].

As first-line investigations, analysis of metabolites in blood, urine and CSF has been used to help in the diagnosis of these patients although they may lack both sensitivity and specificity (e.g. lactate, pyruvate, alanine or organic acids) emphasizing the requirement of better biomarkers [8], [9]. The next step in diagnosis usually involves muscle biopsy investigations (enzymatic activity of the complexes of the respiratory chain). However, this is not always available and in some cases enzyme activity is difficult to interpret.

Fibroblast Growth Factor 21 (FGF-21) has been introduced as a valuable serum biomarker for the detection muscle manifesting mitochondrial disorders [10].

We recently described growth and differentiation factor 15 (GDF-15) as a potential novel biomarker for mitochondrial diseases. GDF-15 mRNA levels were dramatically increased in muscle from patients with *TK2* mutations and the protein was constitutively secreted by skeletal muscle cells. Consistent with this, we found significantly elevated circulating levels of GDF-15 in a small group of patients with a genetic diagnosis of mitochondrial disease [11]. GDF-15 is a cytokine of the Transforming Growth Factor β (TGF-β) superfamily which is expressed mainly in placenta, kidney, liver, lung, pancreas and prostate [12], [13], [14]. This cytokine has an essential role in regulating the cellular response to stress signals and inflammation and has been related with suppression of inflammation in early pregnancy, tumorigenic processes and cardiovascular diseases where is produced by cardiac myocytes in response to ischemia, nitrosative or oxidative stress and angiotensin II [15]. In the CNS, GDF-15 is expressed in the choroid plexus and acts as a potent neurotrophic factor for motor and sensory neurons [16].

With the purpose to evaluate the diagnostic application of GDF-15 we studied its circulating levels in children with mitochondrial encephalomyopathies and other non-mitochondrial neuromuscular diseases and correlated its levels with FGF-21 and various metabolites and clinical signs. We also investigated mRNA expression and protein secretion for both factors in murine and human myotubes under different conditions.

## Materials and Methods

### Ethical statement

The study was approved by the Ethical Committee of the Hospital San Joan de Déu and samples from patients and controls were obtained according to the Helsinki Declarations of 1964, as revised in 2001. Written informed consent for genetic analysis was obtained from patients or their parents/guardians.

### Patients

For this study we included 48 patients for whom we had sufficient clinical, biochemical, and histopathological information to allow us to reach a diagnosis of mitochondrial disease. They were structured into three groups according to Morava criteria. Briefly, this is a scoring system which takes into account clinical (skeletal system, central nervous system, multiorgan disease), metabolic (e.g. elevated lactate and alanine), imaging (e.g. Leigh syndrome lesions on brain MRI) and morphological (e.g. presence of ragged-red and cytochrome c oxidase negative fibers) parameters. Depending on the total score obtained patients are classified with a “definitive”, “probable”, “possible” or “unlikely” mitochondrial disorder [4].

Group 1 consisted of patients with molecularly confirmed mutations in mitochondrial or nuclear DNA (n° patients/samples = 16/22; age range 1–16 yrs). Group 2 were patients with definitive mitochondrial disease following the Morava criteria but no genetic diagnosis (n° patients/samples = 15/20; age range 1 month to 17 yrs) and Group 3 were patients with a probable diagnosis of mitochondrial disease according to the same criteria and also without genetic confirmation (n° patients/samples = 17/17, age; range 1 month to 16 yrs). We collected data regarding age at onset, diagnosis, involvement of skeletal muscle, central nervous system, heart, liver and kidney. Patient information with the corresponding levels of circulating GDF-15 and FGF-21 are detailed in S1 Table.

We also studied a group of patients with non-mitochondrial myopathies, Group 4 (n° patients/samples = 19/19; range 2 months to 18 years) (S1 Table). We selected an age-matched pediatric cohort (n° patients/samples = 33/33; age range 1 month to 18 years) as the control group (Group 5). All controls were healthy subjects who came to the hospital for minor medical interventions.

### Samples

Serum and plasma samples were collected in fasting conditions, separated and stored at -80°C until the moment of the analysis.

### Biochemical analysis

Routine biochemical parameters (blood count, ions, glucose, insulin, hepatic and renal function, lipid and iron metabolism, lactate and pyruvate) were performed by standardized automatic analysis. Plasma amino acids (alanine) were measured by ion exchange chromatography with nynhydrin derivatization and organic acids by gas-chromatography mass spectrometry [17].

GDF-15 and FGF-21 levels were quantified in serum or plasma samples and cell conditioned medium using human or mouse GDF-15 quantikine ELISA kit (R&D Biosystems) and FGF-21 ELISA KIT (Millipore) according to the manufacturer’s instructions.

### Molecular studies

In patients from group 1, the molecular studies were performed for identification of pathogenic mutations in mitochondrial or nuclear DNA. For this, genomic DNA was extracted from different biological samples (blood, muscle or urine) with standard procedures and molecular analyses in mitochondrial or nuclear DNA were performed according to the clinical and biochemical phenotype of the patients (O'Callaghan et al., 2014). In particular, point mutations in mtDNA associated to MELAS and NARP, were determined by PCR-RFLP (PCR- restriction fragment length polymorphism) using the appropriate specific oligonucleotides primers and restriction enzymes for each mutation; the digested products were electrophoresed in agarose gels. Single or multiple deletions in muscle mtDNA were determined by long-range PCR or Southern blot and analysis of mtDNA copy number was performed by quantitative real-time PCR (qRTPCR). Sequencing of TK2, POLG1, and mtDNA was carried out by automated DNA Sanger sequencing methods. The primers and conditions for these analyses are available upon request. Molecular diagnosis data is summarized in S1 Table.

### Histopathological analysis of muscle biopsy

Open muscle biopsy of the right deltoid or left quadriceps muscles were performed. Specimens were either frozen in isopentane cooled by liquid nitrogen for histological and histochemical analysis. Serial frozen sections were stained with standard techniques for hematoxylin and eosin, modified Gomori trichrome, nicotinamide adenine dinucleotide tetrazolium reductase (NADH), succinate dehydrogenase (SDH) and cytochrome c oxidase (COX), combined COX and SDH and Sudan black stain for lipids.

### Cell culture

Mouse myoblastic C2C12 were obtained from the A.T.C.C. (Manassas, VA, U.S.A.). Cells were grown in DMEM (Dulbecco's modified Eagle's medium) containing 10% FBS. At 80% confluence, C2C12 were induced to differentiate into myotubes by changing the medium to DMEM containing 2% HS (horse serum) and DMEM containing 2% FBS respectively. LHCN-M2 human myoblastic cells [Zhu et al. 2007] (a gift from Dr W. Wright, University of Texas Southwestern Medical Center, Dallas, TX, U.S.A.) were maintained in basal medium (DMEM/Medium 199, 4:1 dilution, 0.02 M Hepes, 0.03 μg/ml ZnSO4 and 1.4 μg/ml vitamin B12) containing 15% FBS, 55 ng/ml dexamethasone, 0.01 mg/ml hepatocyte growth factor (Millipore), 0.025 mg/ml FGF (Biological Industries), 50 U/ml penicillin, 50 μg/ml streptomycin and 0.625 μg/ml fungizone. Differentiation of LHCN-M2 cells was induced by culturing in fusion medium (basal medium containing 0.5% FBS, 10 μg/ml insulin, 0.1 mg/ml apo-transferrin and 50 μg/ml dexamethasone) and then in differentiation medium (basal medium containing 0.5% FBS and 55 ng/ml dexamethasone). When indicated cells were treated with the ATP synthase inhibitor oligomycin (0.1 μM) or with the complex III inhibitor antimycin A (10 μM), drug concentrations that did not cause significant cytotoxicity in myotubes (Ribas et al, 2014), and/or 2 mM Trolox (soluble vitamin E derivative), 2 mM ascorbic acid or 2 mM N-acetyl cysteine. For induction of ER stress, myotubes were treated with tunicamycin (1 μM) and thapsigargin (1 μM).

### *In vitro* studies and qRT-PCR

RNA was extracted using Tripure. The levels of FGF21 and myogenin mRNA were determined by qRT-PCR (quantitative reverse transcription—PCR). Reverse transcription was performed in a total volume of 20 μl using random hexamer primers (Applied Biosystems), 0.5 μg of total RNA, and the corresponding TaqMan^®^ Assay-on-demand probes for mouse and human GDF-15 (Mm00442228; Hs00171132), FGF-21 (Mm00840165, Hs00173927) and GRP78 (Mm00517691, Hs99999174) transcripts. qPCR was performed using an ABI/Prism 7500 Sequence Detector System (Applied Biosystems). Each sample was run in duplicate, and the mean value of the duplicate was normalized to that of the 18 S rRNA (Hs99999901) gene using the comparative (2^−ΔC_T_^) method.

### Statistical methods

Statistical analysis was performed using SPSS V.22.0 and PRISM 6.0. Kolmogorov- Smirnov test was applied to determine the distribution of both GDF-15 and FGF-21 values in human samples. Data for GDF-15 and FGF-21 were not normally distributed and for this reason we applied a non-parametric test (U-Mann Whitney) to establish differences in GDF-15 and FGF-21 concentrations between groups and a minimum significance value of p<0.05. The receiving operating characteristics (ROC) curves and area under the curve (AUC) were calculated with PRISM. Spearman Rank Coefficient was used to investigate GDF-15 and FGF-21 association and with other metabolic parameters. Chi-square test was applied to search for categorical associations between GDF-15 and FGF-21 levels and involvement of various organs (S1 Table). In the *in vitro* experimental settings in cells, Student's t test was used to test the level of significance of the differences between means. Pearson's correlation data were determined where indicated.

## Results

### GDF-15 and FGF21 circulating levels

The mean circulating concentration of GDF-15 was almost identical in healthy children (mean = 350.3, SEM = 21 pg/mL) and in myopathic non-mitochondrial controls (349.1 pg/mL) whereas FGF-21 was higher in children with myopathy (136.1 pg/mL) than in unaffected controls (77.6 pg/mL). The range in healthy controls was 155–584 pg/mL for GDF-15 and 21-285pg/mL for FGF-21.

Mean GDF-15 and FGF-21 serum levels were on average 11 times higher in patients (when the three groups were taken together) than in healthy controls (4046, 1492 pg/ml and 885, 156 pg/ml respectively). The mean values and range of GDF-15 and FGF-21 concentrations for each group of patients and controls are summarized in Table 1.

**Table 1:** Serum concentration of GDF-15 and FGF-21 in patients and controls.

**GDF-15**			
Group	Mean (pg/ul)	SEM	Range (pg/ul)
1	7593	3870	205–85252
2	20443	482.7	286–6926
3	1813	788.8	149–13370
4	349.1	32.21	147–809
5	350.3	20.69	155–584
**FGF-21**			
1	966.5	231	25–3623
2	1106	345.7	6–5879
3	522.1	195	17–2658
4	136.1	43.87	30–837
5	77.59	10.3	21–285

SEM: Standard error of the mean. Group 1: patients with molecularly confirmed mitochondrial disease, Group 2: patients with definitive mitochondrial disease, Group 3: patients with probable mitochondrial disease. Group 4: patients with non-mitochondrial myopathy. Group 5: healthy controls.

**Fig 1:**
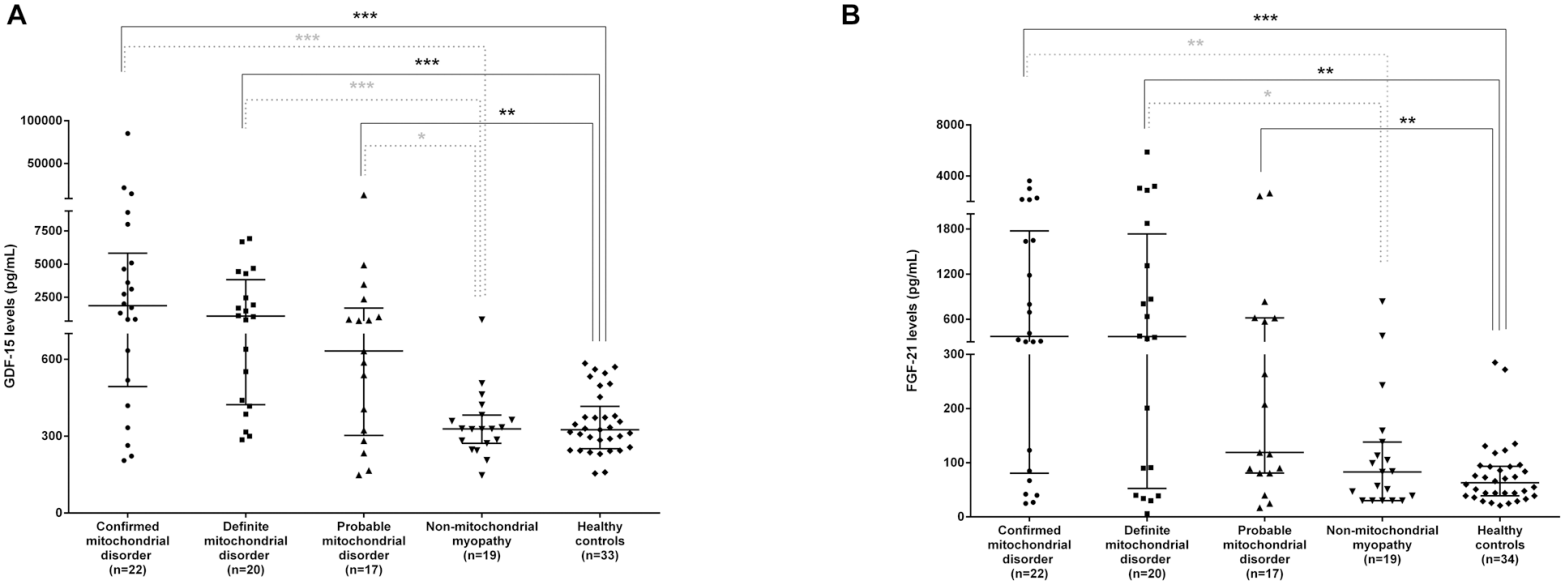
GDF-15 and FGF-21 circulating concentrations. Data are represented as the median +/- the interquartile range. *p< 0.05, **p < 0.01, *** p< 0.001 (U-Mann-Whitney test).

In patients with a diagnosis of mitochondrial disease the levels of both cytokines were significantly elevated relative to healthy controls being the difference more significant in group 1 (confirmed) and 2 (definitive) than in group 3 (probable), (Fig 1). The difference for GDF-15 and FGF-21 when we compared groups 1, 2 and 3 with group 4 (myopathic controls) reached statistical significance in all cases except for the comparison between groups 3 and 4 in the case of FGF-21. Moreover we did not find significant differences between the myopathic and the healthy control groups or between groups 1, 2 and 3 (U-Mann Whitney test applied).

**Fig 2:**
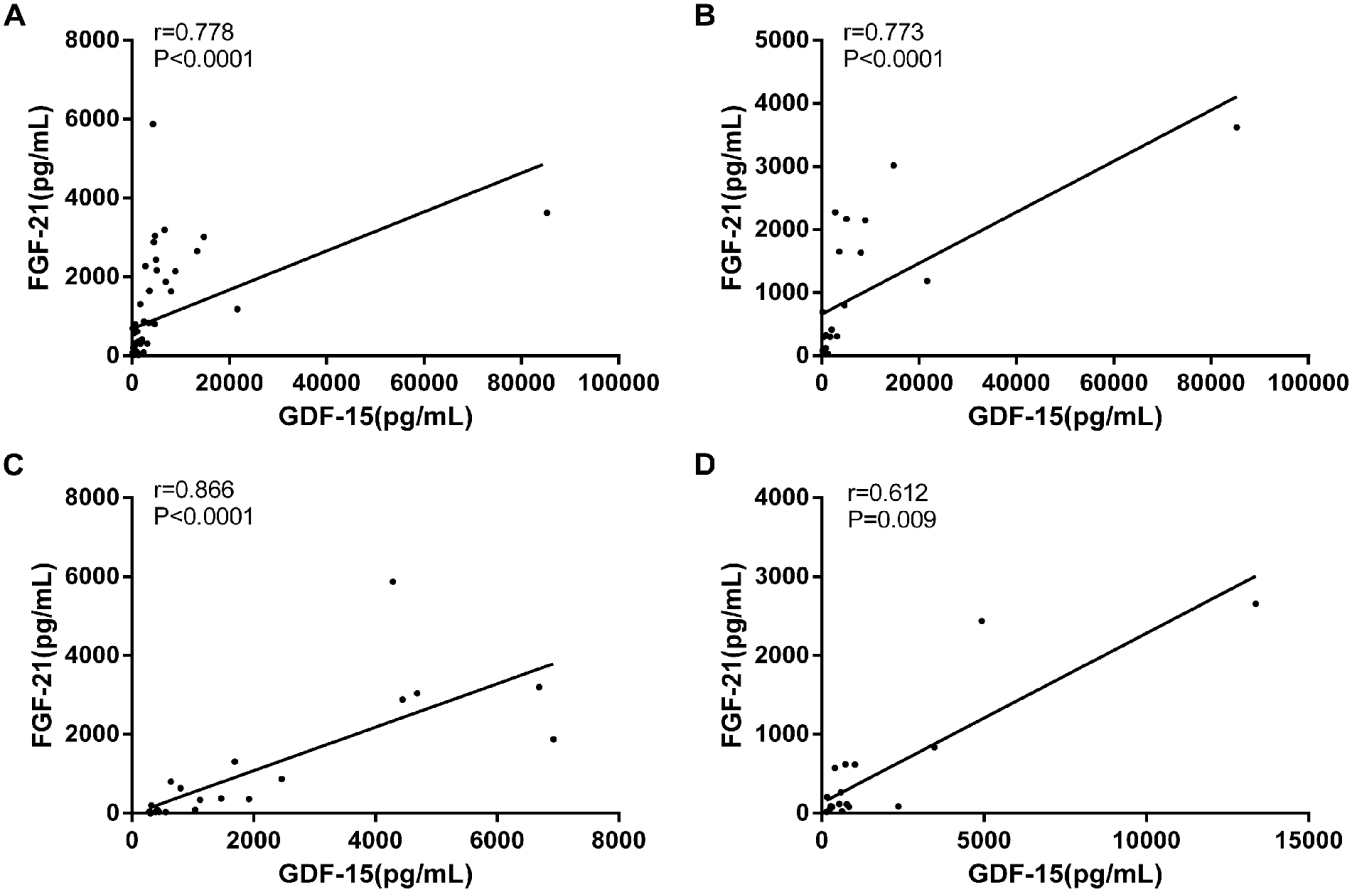
Correlation analysis between GDF-15 and FGF-21. serum levels in samples from all patient groups taken as a whole (A), group 1(B), group 2 (C) and group 3(D).

We observed a positive strong and significant correlation between FGF-21 and GDF-15 values in all patient groups (Spearman’s test), (Fig 2). There was a positive correlation between GDF-15 and alanine transaminase (ALT) and aspartate transaminase (AST) in group 1(AST r = 0.65 p = 0.02; ALT r = 0.7 p = 0.01) and in group 3 (AST r = 0.72 p = 0.008; ALT r = 0.71 p = 0.007) but not in group 2. The remaining of the biochemical parameters analysed (including lactic acid) did not present any association with FGF-21 and GDF-15 values in any of the groups. No significant association was obtained either between FGF-21 and GDF-15 values and the age and gender of the patients.

### Diagnostic performance of GDF-15 and FGF-21

**Fig 3:**
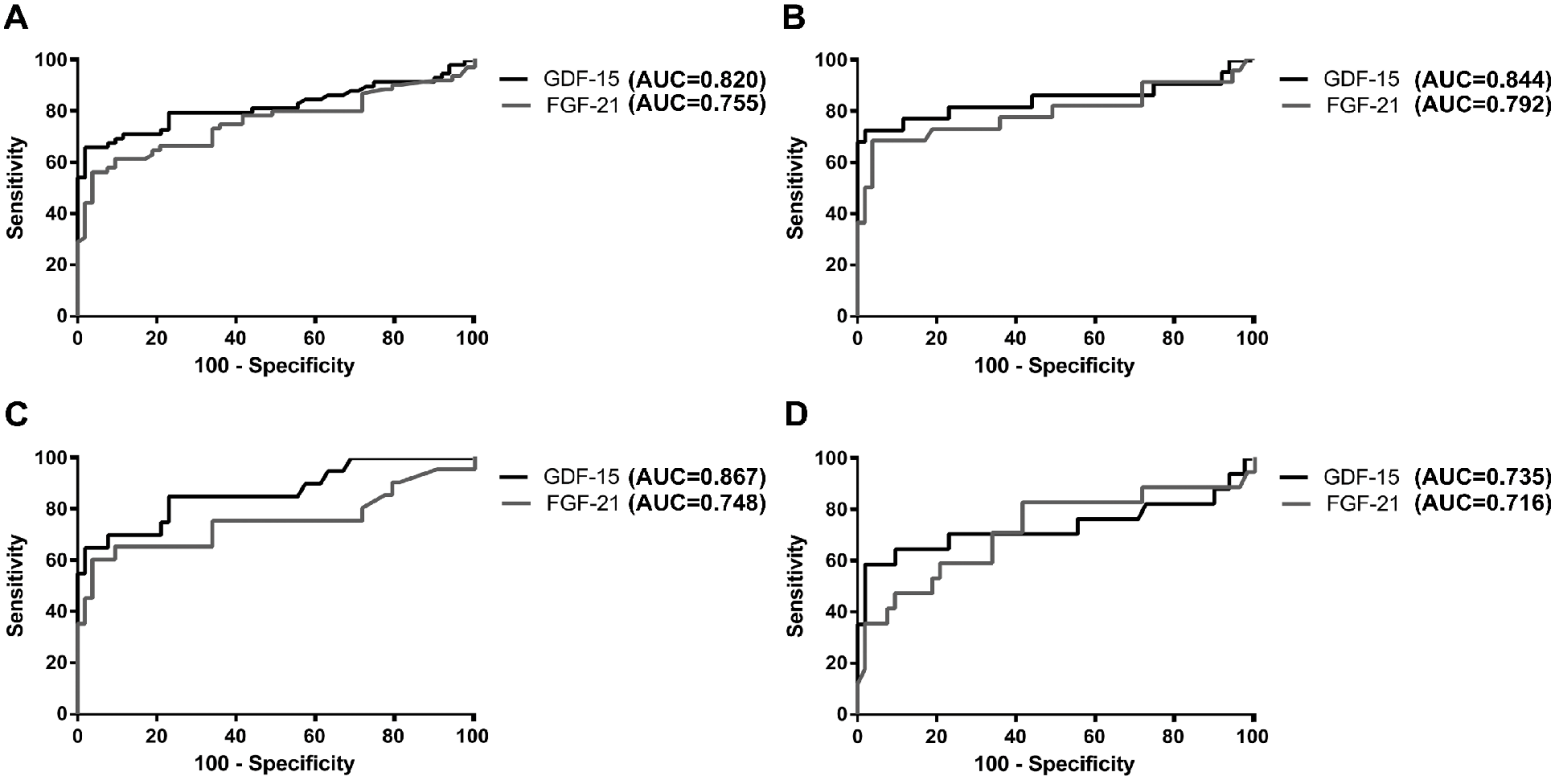
Receiving Operating Curve. analysis for GDF-15 and FGF-21 considering patients in groups 1, 2 and 3 together (A) or patients group 1(B), group 2 (C) and group 3(D) separately.

We performed ROC analysis for GDF-15 and FGF-21 and obtained the area under the curve (AUC). As shown in Fig 3 both factors had a good discriminatory power when all patients were considered together or by groups.

This analysis allowed us to select a clinically useful cut-off value for either factor which would give us an adequate sensitivity and specificity. This value was set at 550pg/ml for GDF-15 and 300 pg/ml for FGF-21. Both values are close to the maximum value in the healthy control group for each soluble factor (see above). The sensitivity, specificity, positive (PPV) and negative (NPV) predictive values for GDF-15 and FGF-21 in each patient group are summarised in Table 2.

**Table 2:** Diagnostic performance of GDF-15 (A) and FGF-21 (B) for a cut-off value of 550pg/ml and 300pg/ml respectively. 95% confidence intervals are indicated in brackets.

**GDF-15**				
Group	Sensitivity	Specificity	PPV	NPV
All	67.8%(54.4%–79.4%)	92.3%(81.5%–97.9%)	93% (80.9%–98.5%)	61.2% (46.2%–74.8%)
Confirmed	72.7% (49.8%–89.2%)	92.3% (81.5%–97.9%)	84.2%(60.4%–96.6%)	83.3%(67.2%–93.6%)
Definitive	70% (45.7%–88.1%)	92.3% (81.5%–97.9%)	82.3%(56.7%–96.2%)	83.3%(67.2%–93.6%)
Probable	58.8%(32.9%–81.6%)	92.3% (81.5%–97.9%)	76.9%(46.2%–94.9%)	81.1% (64.8%–92%)
**FGF-21**				
Group	Sensitivity	Specificity	PPV	NPV
All	52.5% (39.1%–65.7%)	96.2%(87%–99.5%)	100% (89.4%–100%)	56.7% (43.2%–69.4%)
Confirmed	59.1% (36.3% to 79.3%)	96.2%(87%–99.5%)	100% (78.2%–100%)	82.9% (67.9%–92.8%)
Definitive	60%(36.1%–80.9%)	96.2%(87%–99.5%)	100% (73.5%–100%)	80.9%(56.9%–91.4%)
Probable	35.3% (14.2%–61.7%)	96.2%(87%–99.5%)	100% (54.1%–100%)	75.6% (60.5%–87.1%)

**Fig 4:**
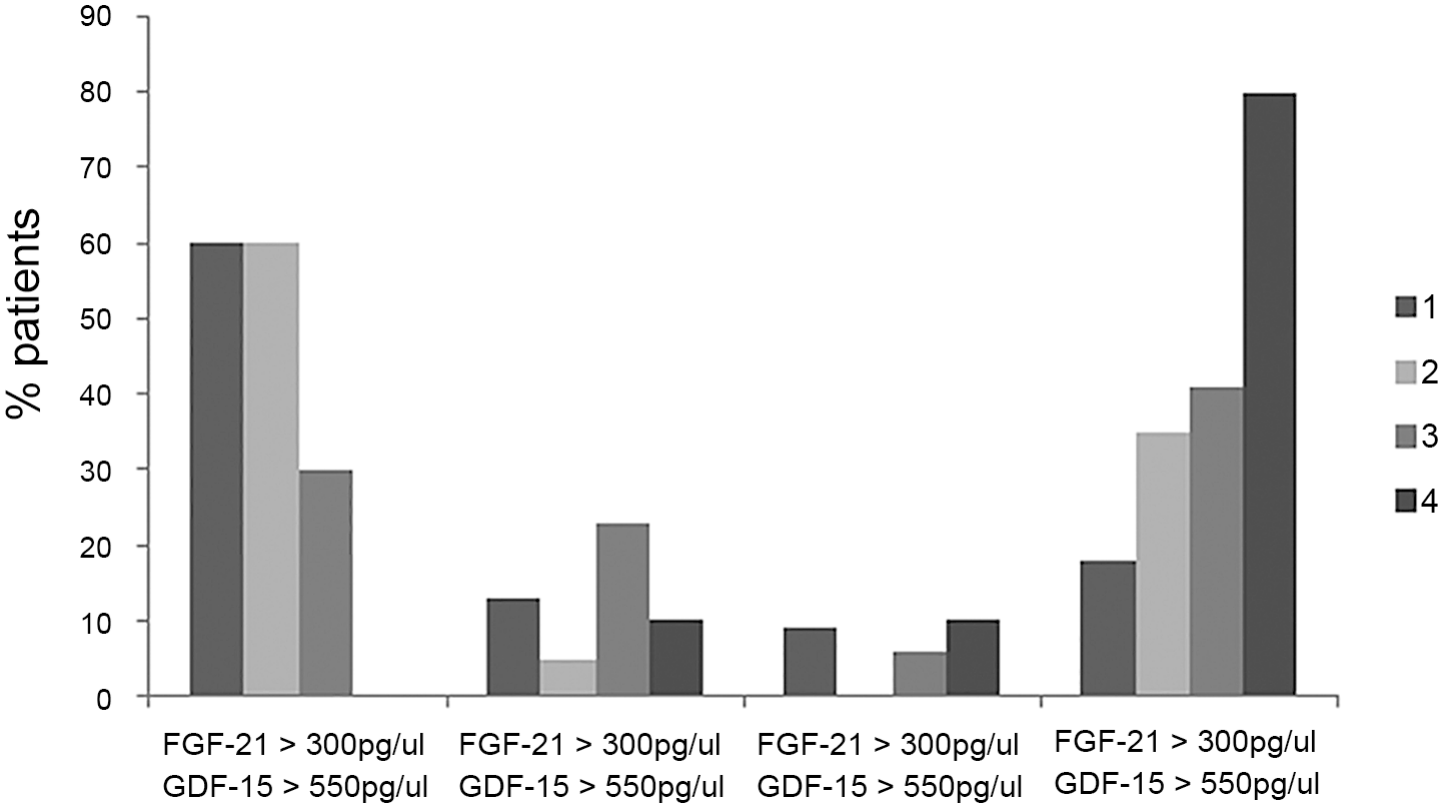
Histogram representing the percentage of patients in each group with both GDF-15 and FGF-21above cut-off values, GDF-15 or FGF-21 elevated or both factors within normal values.

Finally, our data indicated that the combined use of both factors increased the ability of either factor alone to correctly identify patients. The percentage of patients with both GDF-15 and FGF-21 elevated above the cut-off value was higher than the percentage of patients with either elevated GDF-15 or FGF-21 alone. In contrast, the large majority of patients without mitochondrial disease showed values within the normal limit for both factors (Fig 4).

### Experimentally induced mitochondrial dysfunction increases FGF-21 expression and release in muscle cells

**Fig 5:**
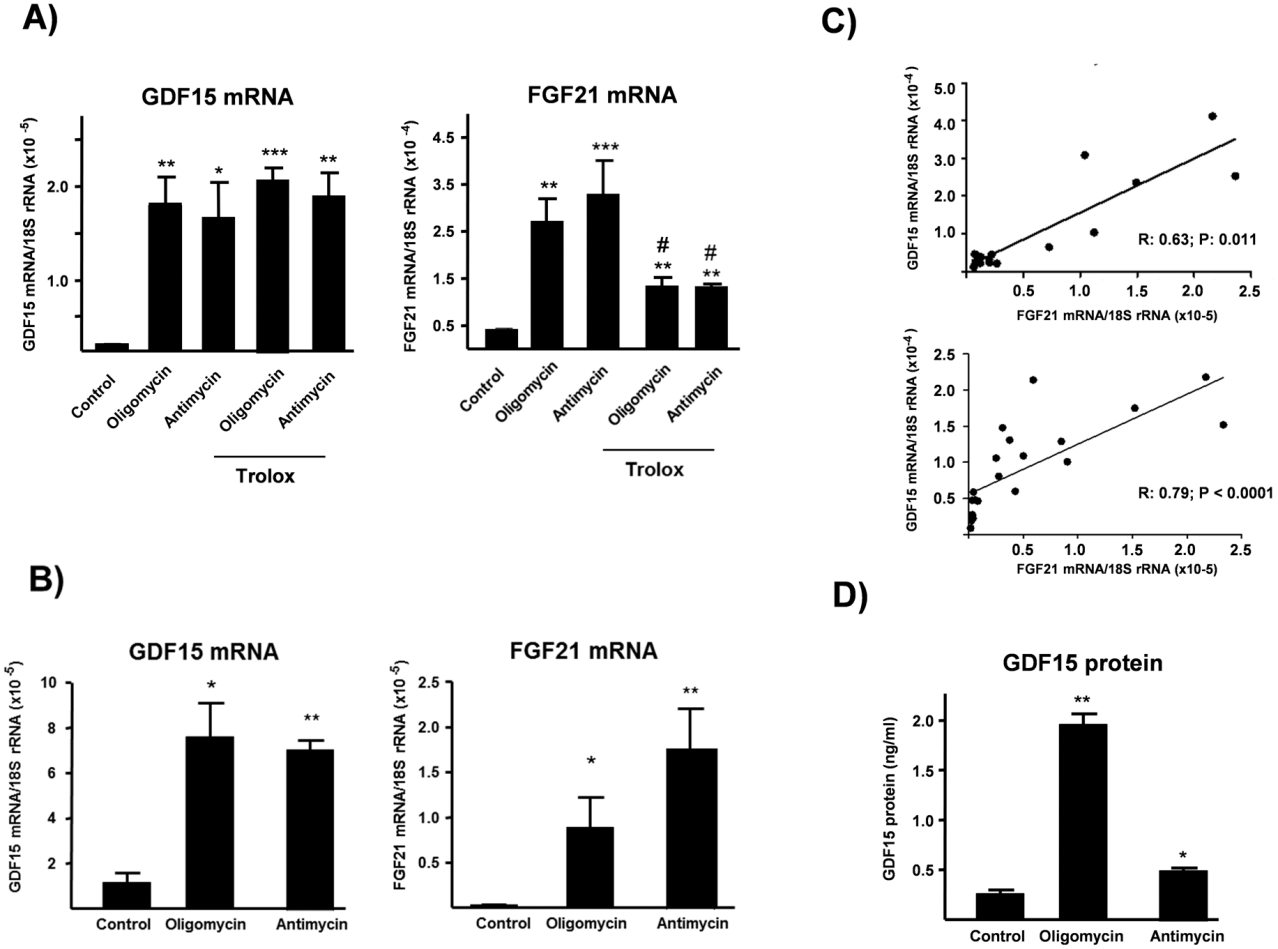
Effects of experimentally induced mitochondrial dysfunction on GDF-15 mRNA expression and GDF15 protein release in muscle cells. GDF15 mRNA and FGF21 mRNA levels in C2C12 myotubes (A) or LHCN myotubes (B), and correlation between GDF-15 mRNA levels and FGF21 mRNA levels (C) in the experimental settings in C2C12 cells (up) and LHCN cells (down). GDF-15 protein concentrations in C2C12 cell culture medium (D). Bars are means ±S.E.M. from 4–6 independent experiments. *p <0.05, **p <0.01, ***p <0.001, relative to untreated controls. ^#^ p <0.05, relative to corresponding condition non-treated with Trolox. R and P values are shown in the correlation panel C.

**Fig 6:**
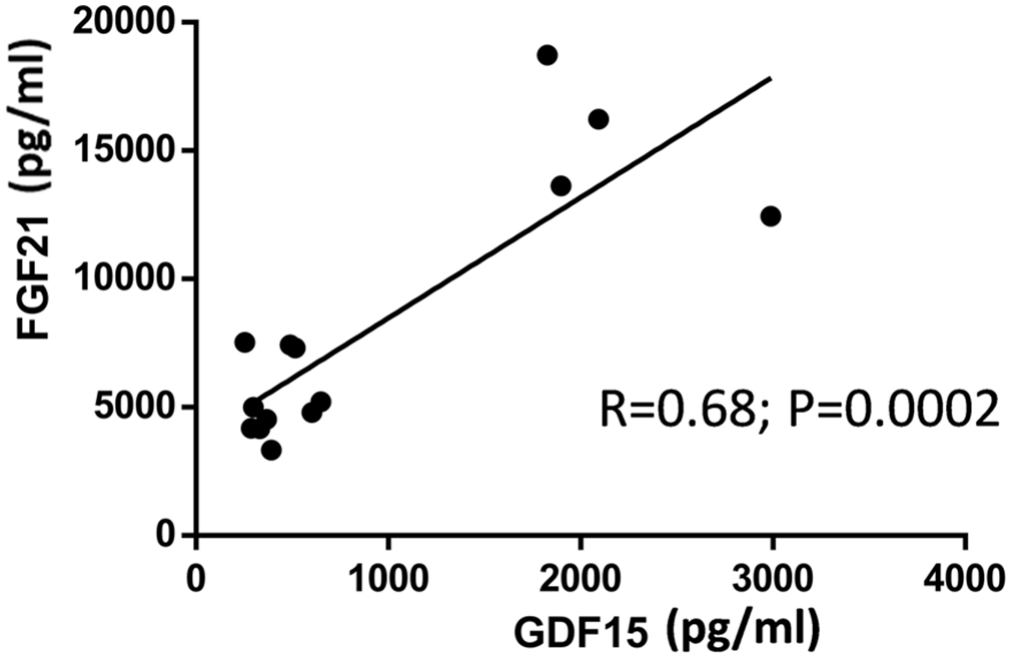
Correlation analysis between GDF-15 and FGF-21 protein levels. in the conditioned medium of C2C12 cells treated with respiratory chain inhibitors.

Considering the above data in patients, we determined whether mitochondrial dysfunction affects GDF-15 gene expression in muscle cells. To this end, we treated C2C12 myotubes with drugs that act at distinct sites of the respiratory chain/oxidative phosphorylation system. Drugs were used at concentrations that did not cause significant cytotoxicity in myotubes and caused an induction of FGF-21 gene expression and release [18]. Treatment with the ATP synthase inhibitor oligomycin (0.1 μM) or with the complex III inhibitor antimycin A (10 μM) for 24 h caused a dramatic induction of GDF-15 gene expression, as well as of FGF-21 gene expression (Fig 5A). In parallel experiments, the effects of oligomycin and antimycin A were determined in human LHCN-M2 myotubes. Similarly to the results obtained in mouse myogenic cells, oligomycin and antimycin A induced a significant increase in GDF15 mRNA expression in human LHCN-M2 myotubes, as well as of FGF21 mRNA (Fig 5B). Correlation analysis settings indicated a highly significant correlation between the extent of GDF15 mRNA expression and FGF21 mRNA expression in cells under the distinct experimental settings both in mouse and humans muscle cells (Fig 5). Treatment of C2C12 myotubes with oligomycin for 24h resulted in a marked increase in GDF15 protein levels released to the cell culture medium (Fig 5) and there was a minor but significant increase due to the treatment with antimycin (Fig 5D). As for mRNA, protein levels of FGF21 and GDF15 released to the cell culture medium in the experimental conditions tested showed a significant correlation (Fig 6). However, no significant effects on GDF15 protein accumulation were found in the LHCN-M2 cell model (data not shown).

Previous studies have shown that reactive oxygen species (ROS) production is involved in the induction of FGF21 expression by experimental mitochondrial dysfunction [18]. Effectively, treatment of myotubes with the ROS scavenger Trolox blunted oligomycin- and antimycin-induced FGF21 expression. In contrast, the induction of GDF-15 by the mitochondrial function inhibitors was insensitive to the presence of the ROS scavenger (Fig 4A). Other ROS scavengers such as ascorbic acid or N-acetyl cysteine were also unable to blunt the induction of GDF-15 expression in response to oligomycin or antimycin (data not shown). Thus, in contrast with FGF-21, intracellular mechanisms other than enhanced ROS induction account for the GDF-15 induction in response to altered mitochondrial function.

We had previously shown that GDF-15 skeletal muscle mRNA levels dramatically increased in patients with mitochondrial disease and that human myotubes constitutively secreted this cytokine [11]. To investigate the regulation of GDF-15 expression we quantified its mRNA during myogenic differentiation in C2C12 and human LHCN-M2 myogenic cells and compared it to myogenin and FGF-21 which are induced during myogenesis [18]. In contrast to myogenin and FGF-21 we did not observe an increase in GDF-15 expression between undifferentiated cells and cells, 1, 3 or 5 days post-differentiation in C2C12 cells, nor in the transition from myoblastic stage to differentiated muscle (myotube) stage in human LHCN-M2 cells (data not shown). We explored whether ER (endoplasmic reticulum stress) was involved in oligomycin- or antimycin A-induced GDF15 gene expression. Induction of ER stress by use of drugs such as tunicamycin or thapsigargin induced strongly the expression of GDF15 and FGF21, indicating the sensitivity of these genes to this pathway of cellular stress (S2 Table). However, we found no change in the expression of the ER-stress-response marker gene GRP78 following exposure of C2C12 myotubes or LHCN-M2 myotubes to oligomycin or antimycin A at the concentrations tested (S3 Table). Thus, although involvement of ER stress in the regulation of GDF15 (and FGF21) cannot be ruled out as involved in the induction of these genes in damaged muscle, mitochondrial-driven pathways of control appear to occur independently from ER stress.

## Discussion

In the present study we have compared circulating GDF-15 and FGF-21 levels in a cohort consisting exclusively of children with a diagnosis of mitochondrial disease which included patients with mutations in both mitochondrial and nuclear DNA.

Just before submission Yatsuga et al. reported elevated GDF-15 levels in a group of patients (mainly adults) with mutations in mitochondrial DNA [19] (KSS, MELAS or Leigh Syndrome). Our work differs from Yatsuga and co-workers work in that we focus exclusively on children under the age of 18 (mean age 6.7 years versus 33 years) and that we have studied patients both with mutations in mitochondrial and nuclear DNA with a wide range of phenotypes. We also decided to include patients without known mutations in either nuclear or mitochondrial DNA but with established clinical and other criteria of mitochondrial disease according to Morava diagnostic score. We thought that given the clinical and genetic heterogeneity of mitochondrial diseases and the difficulty to reach a molecular diagnosis in children it would be more helpful to test GDF-15 in those patients with different degrees of suspicion than only in patients with already known molecular defects. The rationale being that if proven sensitive it would then help selecting patients for further biochemical and genetic analysis reducing the time and cost of the diagnostic work up. Lastly, we provide experimental evidence that mitochondrial dysfunction in skeletal muscle cells leads to GDF-15 induction.

Our results indicate that GDF-15 is a sensitive and specific biomarker to guide the diagnosis of this group of complex genetic diseases. Furthermore we show that the combined use of GDF-15 and FGF-21 is more efficient in identifying patients than either factor alone. This strategy would be useful for example to select patients for comprehensive genetic analysis, which is still expensive and not available in all centers.

GDF-15 has been studied before in the context of cancer, obesity, type II diabetes, malaria and heart disease [20], [21], [22], [15]. In those studies GDF-15 values in the control groups were similar to the ones we found in the present study (c.300pg/ml) in both children and adults supporting our finding that GDF-15 does not correlate with age.

In the present study, GDF-15 average circulating concentration in mitochondrial patients was similar to our previous data [11] and other recent publications [19][23].

Amongst patients with the highest levels of GDF-15 were children with mutations in *TK2*, patients with MELAS and the common mutations in the *MT-TL1* gene and patients with deletions in mtDNA including one patient with Pearson Syndrome and two patients with KSS. Out of patients with KSS the one with kidney involvement (P25) had the higher levels of GDF-15 (and FGF-21) suggesting that kidney may be an important source of both factors. In fact, GDF-15 is expressed in the collecting ducts in the kidney [24] and there are publications that associate GDF-15 with kidney dysfunction in mice [12] and in some pathologies such as diabetic nephropathy [25] and in patients undergoing coronary artery bypass grafting [26]. It is worth noting that patients with multisystemic mitochondrial disease with kidney and or CNS involvement had markedly elevated levels of GDF-15 (P19, P39 and P41).

GDF-15 is expressed in the brain where it is produced and secreted into the CSF by the chroroid plexus cells [16]. Thus, it would be interesting to investigate GDF-15 concentrations in CSF.

Five patients with mutations in mtDNA or nuclear DNA showed GDF-15 values below the cut-off value of 550pg/ml. Four of these also had normal FGF-21 concentrations. These were patients with a mainly neurological presentation including 2 patients with PDHA1 deficiency, a patient with a severe encephalopathy and mutations in the *GFM1* gene [27], a patient with NARP (neuropathy, ataxia and retinitis pigmentosa) and a patient with mutations in *OPA1*. The reason of these normal values is unclear and requires further investigation, but it may be explained, in the case of mitochondrial DNA mutations, because of the phenomenon of heteroplasmy, or in the case of nuclear DNA mutations, by the tissue specificity of the phenotype.

Regarding FGF-21 patients with values within the normal range have already been described [10]. Amongst patients with genetically confirmed mitochondrial disease (group 1) we found 3 who had normal levels of FGF-21 but elevated GDF-15 and they all had the common MELAS mutation. Thus, GDF-15 may be particularly helpful in this group of patients. Two of them (P10 and P11) are two sisters from a family with several affected members with MELAS [28]. P10 has migraines as the only symptom and P11 is currently asymptomatic. Thus, GDF-15 may be able to detect early symptoms and sub-clinical presentations although in a recent report GDF-15 did not correlate with disease progression in adults bearing the m.A3243>G mutation [29].

Some patients had more than one sample re-tested at different time points. In those patients levels of GDF-15 tended to increase with time or remained unchanged. For example, in P8 with mutations in *TK2* and a severely progressive clinical course, GDF-15 levels increased by 1.5 fold in a 6 months period. This patient was started on nucleotide replacement therapy [30] and a third serum sample taken 7 months after start of treatment showed an important decrease in GDF-15 levels. We are currently investigating more patients with mutations in *TK2* under treatment. These data suggest that GDF-15 may be useful to monitor disease progression and response to treatment in mitochondrial myopathies.

GDF-15 AUC values were slightly higher than those for FGF-21, consistent with recent reports. All groups presented high AUC values (above 0.8) confirming that GDF-15 is able to discriminate properly between a patient and a healthy individual. In our study, however the AUC for GDF-15 and FGF-21 were lower than reported previously [10],[19]. This may be due to the fact that our patient cohort is more heterogeneous than in the above mentioned studies and to the sample size of each sub-group. We believe however that it was important to study patients with a reasonable diagnostic criteria of mitochondrial disease (according to clinical, pathological, radiological and biochemical parameters) even if genetic confirmation was pending because this situation is closer to daily clinical practice. In this way we were able to assess whether GDF-15 was useful to help clinicians to prioritize patients for further genetic analysis and or muscle biopsy.

We did not find significant correlations between GDF-15 and FGF-21 and other metabolites including lactate and pyruvate. In fact, in all patient groups we found that a proportion of patients (between 16% and 29% depending on the group) had normal lactate levels but both GDF-15 and FGF-21 elevated. This suggests that these factors are more sensitive than lactate. We did not find any positive associations either between GDF-15 or FGF-21 levels and involvement of skeletal muscle, CNS, heart or kidney (S1 Table).

The induction of GDF-15 by mitochondrial respiratory chain inhibitors in myogenic cells that we have observed in the present study indicates that, similarly to FGF21, disturbing mitochondrial function is a powerful stimulus for the expression and release of both factors. This observation provides a strong mechanistic support to the common induction of GDF-15 and FGF21 levels in patients. For FGF21, reactive oxygen species production by mitochondria play a main role in the induction of FGF21 gene expression, and anti-oxidants can block it. In contrast, anti-oxidants are unable to block the GDF-15 induction elicited by mitochondrial inhibitors. These findings do not rule out the involvement of oxidative stress in the regulation of GDF15 gene in some cell types or under distinct challenges [15] but indicate that the intracellular mechanisms of “mitochondrial retrograde signaling” to the FGF21 and GDF15 genes are distinct and, for GDF15 may involve processes other than ROS production in muscle mitochondria such as ER stress [31]. However, despite we found that ER stress signaling could induce GDF15 in myotubes, the fact that mitochondrial-driven drugs at the concentrations used induce GDF15 expression but not marker genes of ER stress, suggest the existence of additional, mitochondria dependent, pathways of GDF15 gene regulation.

To summarise, in the present study we have shown that GDF-15 is a valuable diagnostic marker to aid in the diagnostic work-up of children with a suspicion of mitochondrial disease. The combined analysis of GDF-15 and FGF-21 allows for a more guided selection of patients for further biochemical and genetic analysis. GDF-15 (and FGF-21) may also be applied as surrogate markers to monitor the progression of these diseases or the effect of a treatment. Further studies are necessary to understand the mechanisms regulating GDF-15 under conditions of mitochondrial dysfunction and cellular stress.

## Supporting Information

S1 Table Summary of patients characteristics and GDF-15 and FGF-21 concentrations. (DOCX)

S2 Table Effects of tunicamycin and thapsigargin on GDF-15 and FGF21 mRNA expression in differentiated myotubes. (DOCX)

S3 Table Effects of oligomycin, antimycin A, tunicamycin and thapsigargin on GPR78 mRNA expression in differentiated myotubes. (DOCX)
