# Correction: GDF-15 Is Elevated in Children with Mitochondrial Diseases and Is Induced by Mitochondrial Dysfunction

**DOI:** 10.1371/journal.pone.0155172

**Published:** 2016-05-04

**Authors:** The PLOS ONE Staff

There is an error in Table 1. The value listed under GDF-15, Mean (pg/ul), Group 2, should be 2043, not 20443. Please see the correct Table 1 and its caption below.

**Table 1:** Serum concentration of GDF-15 and FGF-21 in patients and controls.

**GDF-15**			
Group	Mean (pg/ul)	SEM	Range (pg/ul)
1	7593	3870	205–85252
2	2043	482.7	286–6926
3	1813	788.8	149–13370
4	349.1	32.21	147–809
5	350.3	20.69	155–584
**FGF-21**			
1	966.5	231	25–3623
2	1106	345.7	6–5879
3	522.1	195	17–2658
4	136.1	43.87	30–837
5	77.59	10.3	21–285

SEM: Standard error of the mean. Group 1: patients with molecularly confirmed mitochondrial disease, Group 2: patients with definitive mitochondrial disease, Group 3: patients with probable mitochondrial disease. Group 4: patients with non-mitochondrial myopathy. Group 5: healthy controls.

Fig 4 appears incorrectly in the published article. Please see the correct Fig 4 and its caption below.

**Fig 4:**
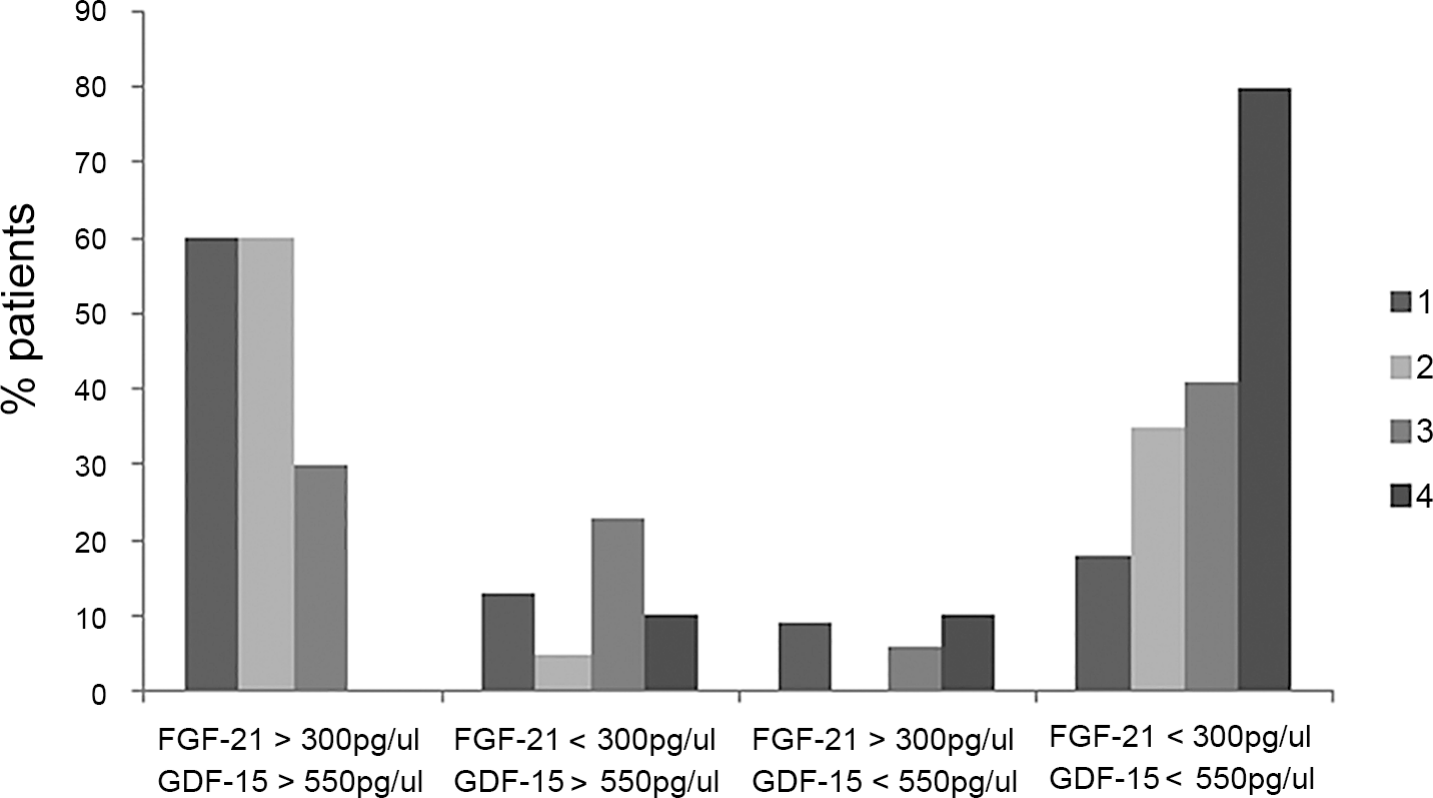
Histogram representing the percentage of patients in each group with both GDF-15 and FGF-21above cut-off values, GDF-15 or FGF-21 elevated or both factors within normal values.

The publisher apologizes for the errors.
