# Supplementary material for: GDF-15 Is Elevated in Children with Mitochondrial Diseases and Is Induced by Mitochondrial Dysfunction
Source: PLoS One. 2016 Feb 11;11(2):e0148709. doi: 10.1371/journal.pone.0148709 (PMC4750949; doi:10.1371/journal.pone.0148709)
Supplement: S1 Table — (DOCX) [file pone.0148709.s001.docx]

| **Supp. Table 1: Patients characteristics and GDF-15 and FGF-21 concentrations** | | | | | | |  |  |  |  |  |
| --- | --- | --- | --- | --- | --- | --- | --- | --- | --- | --- | --- |
|  | Age at onset | Age at sampling | Diagnosis | Skm | CNS | Heart | Liver | Kidney | DNA diagnosis | **GDF-15 (pg/ml)** | **FGF21**  **(pg/mL)** |
| **Genetically Confirmed Mitochondrial Disease** | | | |  |  |  |  |  |  |  |  |
| P1 | 4 y | 13y4m  14y10m | MELAS | Yes | No | Yes | No | No | m.3243A>G (tRNA^Leu(UUR)^) (75% muscle, 75% urine, 10% buccal mucose) | 4626  8898 | 800  2146 |
| P2 | 12 m | 1y10m  3y6m  4y5m | Pearson Syndrome | No | No | No | No | No | m.10600_14603del4004 (90% muscle) | 1721  3609  2740 | 302  1650  2274 |
| P3 | 7 y | 10y | MELAS | Yes | Yes | ND | ND | ND | m.3243A>G (tRNA^Leu(UUR)^) | 1308 | 25 |
| P4 | 2 y | 7y | MDS (TK2) | Yes | No | ND | ND | ND | *TK2*: c.[542C>T];[542C>T], p.[(A181V)];[(A181V)] | 3114 | 311 |
| P5 | 2 y | 2y | MDS (TK2) | Yes | No | ND | ND | ND | *TK2*: c.[542C>T];[542C>T], p.[(A181V)];[(A181V)] | 8000 | 1636 |
| P6 | 10 y | 13y | PEO | Yes | No | No | No | No | m.8483_13459del4977 (50% muscle, 65% urine) | 841 | 329 |
| P7 | 6 y | 15y | POLG1 Deficiency | Yes | Yes | No | Yes | No | *POLG*: c.[911T>G];[911T>G], p.[(Leu304Arg)];[(Leu304Arg)] | 85252 | 3623 |
| P8 | 16 m | 1y9m  2y3m  3y2m | MDS (TK2) | Yes | No | No | No | No | *TK2*: c.[623A>G];[388C>T], p.[(Tyr208Cys)];[(Arg130Trp)] (mtDNA 85% depletion muscle) | 14756  21648  5093 | 3017  1186  2169 |
| P9 | Neonatal | 3y9m  4y10m | GFM1 Deficiency | No | Yes | No | No | No | *GFM1*: c.[104delA];[2011C>T], p.[(Ser467fs)];[(Arg671Cys)] | 518-419 | 40  302 |
| P10 | ND | 11y | MELAS | No | Yes | No | No | No | m.3243A>G (tRNA^Leu(UUR)^) (3% blood, 75% urine, 31% buccal mucose) | 634 | 67 |
| P11 | ND | 9y | MELAS | No | No | No | No | No | m.3243A>G (tRNA^Leu(UUR)^) (47% blood, <5% urine, 32% buccal mucose) | 829 | 123 |
| P12 | Neonatal | 5 y | OPA1 Deficiency | No | Yes | ND | No | No | *OPA1*: c.[1710T>G], p.[(Phe570Leu)] | 333 | 27 |
| P13 | 2 y | 16y | PDH Deficiency | Yes | Yes | No | No | No | *PDHA1*: c.[1143-1144ins24] | 222 | 85 |
| P14 | 18 m | 5y | PDH Deficiency | Yes | Yes | No | No | No | *PDHA1*: c.[787C>G], p.[(Arg263Gly)] | 205 | 695 |
| P15 | 15 m | 7y | NARP | Yes | Yes | ND | No | No | m.9176T>C (ATPase 6) (99,5% blood, 99% bucal mucose, 99,5% urine) | 264 | 42 |
| P16 | 14 m | 3y | MDS (TK2) | Yes | No | No | No | No | *TK2*: c.[360_361delGCinsAA];c.[575G>A], p.[(His121Asn)];[(Arg192Lys)] | 2011 | 415 |
| **Definitive Mitochondrial Disease** | | | |  |  |  |  |  |  |  |  |
| P17 | Neonatal | 3y | Mitochondrial Encephalomyopathy | Yes | Yes | No | No | No | ND | 552 | 30 |
| P18 | 9 m | 3y | Mitochondrial Encephalomyopathy | Yes | Yes | No | No | No | ND | 639 | 806 |
| P19 | 13 m | 3y - 6y | Multisystem Mitochondrial Failure | No | Yes | No | No | Yes | ND | 6689  4286 | 5879  3196 |
| P20 | Neonatal | 4y  4y11m | Mitochondrial Encephalopathy | No | Yes | No | No | No | ND | 1039  797 | 91  638 |
| P21 | Neonatal | 1m | Multisystem Mitochondrial Failure | No | Yes | No | No | No | ND | 1686 | 1312 |
| P22 | 8 m | 3y7m 3y10m | Mitochondrial Encephalopathy | Yes | Yes | No | ND | No | ND | 1117  1464 | 341  379 |
| P23 | 3 y | 12y | Leigh Syndrome | No | Yes | No | No | No | ND | 417 | 90 |
| P24 | Neonatal | 14y  17y | Barth Syndrome | ND | ND | ND | ND | ND | ND | 4446  4685 | 2884  3043 |
| P25 | 9 y | 16y | Kearn Sayre Syndrome | Yes | Yes | No | No | Yes | ND | 6926 | 1876 |
| P26 | Neonatal | 4y | Mitochondrial Encefalopathy | Yes | Yes | No | No | No | ND | 316 | 201 |
| P27 | 3 y | 8y7m 9y4m | Leigh Syndrome | Yes | Yes | No | No | No | ND | 300  386 | 6  39 |
| P28 | Neonatal | 1y | Mitochondrial Encephalopathy | No | Yes | No | No | No | ND | 440 | 34 |
| P29 | Neonatal | 10m | Leigh Syndrome | Yes | Yes | Yes | No | No | ND | 1923 | 362 |
| P30 | 6 y | 11y | Kearn Sayre Syndrome | Yes | Yes | Yes | No | No | ND | 2462 | 869 |
| P31 | Neonatal | 4y | Mitochondrial Encephalomyopathy | Yes | Yes | ND | No | No | ND | 286 | 40 |
| **Probable Mitochondrial Disease** | | | |  |  |  |  |  |  |  |  |
| P32 | Neonatal | 3 y | Mitochondrial Encephalopathy | No | Yes | ND | No | No | ND | 632 | 25 |
| P33 | 19 m | 4 y | Mitochondrial Encephalomyopathy | Yes | Yes | No | No | No | ND | 539 | 119 |
| P34 | Neonatal | 15 y | Multisystem Mitochondrial Failure | Yes | Yes | No | No | No | ND | 323 | 81 |
| P35 | Neonatal | 13y11m 14y5m | Mitochondrial Encephalopathy | Yes | Yes | No | No | No | ND | 406  733 | 574  621 |
| P36 | Neonatal | 10m | Mitochondrial Encephalopathy | No | Yes | No | No | No | ND | 834 | 81 |
| P37 | 6 y | 16y | Mitochondrial Encephalopathy | No | Yes | ND | No | No | ND | 282 | 90 |
| P38 | 2 y | 13y | Multisystem Mitochondrial Failure | No | Yes | No | No | Yes | ND | 166 | 208 |
| P39 | Neonatal | 12y | Multisystem Mitochondrial Failure | No | Yes | No | No | No | ND | 13370 | 2658 |
| P40 | Neonatal | 1m | Mitochondrial Encephalopathy | No | Yes | No | No | No | ND | 771 | 116 |
| P41 | 15m | 3y | Mitochondrial Nephropathy | No | No | No | No | Yes | ND | 4931 | 2438 |
| P42 | Neonatal | 4y | Multisystem Mitochondrial Failure | No | No | No | No | No | ND | 2277 | 124 |
| P43 | 3 m | 4m | Multisystem Mitochondrial Failure | No | Yes | No | No | No | ND | 1019 | 618 |
| P44 | 8 m | 2y | Mitochondrial Nephropathy | No | No | No | No | Yes | ND | 2361 | 89 |
| P45 | 20 m | 7y | Mitochondrial Encephalopathy | No | Yes | No | No | No | ND | 234 | 40 |
| P46 | Neonatal | 1y | Mitochondrial Encephalomyopathy | ND | ND | ND | ND | ND | ND | 3475 | 837 |
| P47 | Neonatal | 10m | Mitochondrial Encephalopathy | No | Yes | No | No | No | ND | 588 | 264 |
| P48 | 18 m | 5y | Mitochondrial Encephalopathy | Yes | No | No | No | No | ND | 149 | 17 |
| **Non-mitochondrial Myopathy** | | | |  |  |  |  |  |  |  |  |
| P49 | Neonatal | 11y | UCMD | Yes | No | No | No | No | *COL6A1: c.* [717+4A>G], p.[ (271Arg>X)] | 327 | 243 |
| P50 | Neonatal | 8y | UCMD | Yes | No | No | No | No | *COL6A1: c.* [717+4A>G], p.[ (271Arg>X)] | 334 | 837 |
| P51 | Neonatal | 6y | UCMD | Yes | No | No | No | No | *COL6A1:* c.[877G>A], p.[(Gly293Arg)] | 147 | 99 |
| P52 | Neonatal | 5y | UCMD | Yes | No | No | No | No | ND | 359 | 30 |
| P53 | Neonatal | 6y | UCMD | Yes | No | No | No | No | *COL6A1*: c.[868 G>A], p. [(Gly290Arg)] | 327 | 113 |
| P54 | Neonatal | 19y | UCMD | Yes | No | No | No | No | *COL6A1* (details not available) | 506 | 159 |
| P55 | Neonatal | 16y | UCMD | Yes | No | No | No | No | *COL6A1* (details not available) | 247 | 105 |
| P56 | Neonatal | 8y | UCMD | Yes | No | No | No | No | *COL6A1:* c.[877G>A], p.[(Gly293Arg)] | 272 | 138 |
| P57 | 9y | 17y | BM | Yes | No | No | No | No | *COL6A1:* c.[877G>A], p.[(Gly293Arg)] | 206 | 84 |
| P58 | 5y | 8y | BMD | Yes | No | No | No | No | *DMD*: c.[3850C>T], p. [(1284Glu>X)] | 382 | 57 |
| P59 | 3y | 5y | DMD | Yes | Yes | Yes | No | No | *DMD*: c.[3578T>A], p. [(1193Leu>X)] | 329 | 39 |
| P60 | 3y | 4y | DMD | Yes | Yes | No | No | No | *DMD* (details NA) | 422 | 30 |
| P61 | 3y | 4y | DMD | Yes | Yes | Yes | No | No | *DMD*: c.[2733insG], p [925 | 282 | 30 |
| P62 | 3y | 12y | DMD | Yes | Yes | No | No | No | *DMD* (details NA) | 363 | 83 |
| P63 | 3y | 3y | DMD | Yes | No | No | No | No | *DMD* (details NA) | 244 | 47 |
| P64 | 3y | 6y | DMD | Yes | Yes | No | No | No | *DMD*: c.[583C>T], p. [(195Arg>X)] | 286 | 380 |
| P65 | 3 y | 8y | DMD | Yes | Yes | Yes | No | No | *DMD* (details NA) | 328 | 30 |
| P66 | Neonatal | 1y | CM with cores | Yes | No | No | No | No | ND | 462 | 51 |
| P67 | Neonatal | 1y | CMD | Yes | No | Yes | No | No | *LMNA:* c[745C>T],p. [(249Arg>Trp)] | 809 | 30 |

Neonatal: within 1st month of life; NA= not available; ND= not determined; y=years; m=months; Skm=skeletal muscle; DMD=Duchenne Muscular Dystrophy; BMD= Becker Muscular Dystrophy; UCMD: Ullrich Congenital Muscular Dystrophy; BM: Bethlem Myopathy; CM: congenital Myopathy; CMD: Congenital Muscular Dystrophy
