# Supplementary material for: GDF-15 Is Elevated in Children with Mitochondrial Diseases and Is Induced by Mitochondrial Dysfunction
Source: PLoS One. 2016 Feb 11;11(2):e0148709. doi: 10.1371/journal.pone.0148709 (PMC4750949; doi:10.1371/journal.pone.0148709)
Supplement: S2 Table — (DOCX) [file pone.0148709.s002.docx]

**S2 Table. Effects of tunicamycin and thapsigargin on GDF-15 and FGF21 mRNA expression in differentiated myotubes.**

FGF21 mRNA GDF15 mRNA

(fold- induction vs controls) (fold- induction vs controls)

___________________________________________________________________________

C2C12 myotubes:

Tunicamycin (1 µM) 18 + 1*** 22 + 4 ***

Thapisgargin (1 µM) 25+ 4*** 21 + 2***

LHCN-M2 myotubes:

Tunicamycin (1 µM) 78 + 3*** 10 + 1 ***

Thapisgargin (1 µM) 99 + 6*** 11 + 1***

Differentiated myotubes were treated with drugs during 24h. Dara are means + SEM of three independent experiments. ***P < 0.001 in comparison with control, untreated, cells.
