# Supplementary material for: GDF-15 Is Elevated in Children with Mitochondrial Diseases and Is Induced by Mitochondrial Dysfunction
Source: PLoS One. 2016 Feb 11;11(2):e0148709. doi: 10.1371/journal.pone.0148709 (PMC4750949; doi:10.1371/journal.pone.0148709)
Supplement: S3 Table — (DOCX) [file pone.0148709.s003.docx]

**S3 Table. Effects of oligomycin, antimycin A, tunicamycin and thapsigargin on GPR78 mRNA expression in differentiated myotubes.**

GPR28 mRNA

(fold- induction vs controls) ___________________________________________________________________________

C2C12 myotubes:

Oligomycin (0.1 µM) 1.3 + 0.2 NS

Antimycin A (10 µM) 1.4 + 0.4 NS

Tunicamycin (1 µM) 18 + 2***

Thapisgargin (1 µM) 22 + 2***

LHCN-M2 myotubes:

Oligomycin (0.1 µM) 1.2 + 0.3 NS

Antimycin A (10 µM) 1.7 + 0.4 NS

Tunicamycin (1 µM) 36 + 3***

Thapisgargin (1 µM) 41 + 2***

___________________________________________________________________________

Differentiated myotubes were treated with drugs during 24h. Dara are means + SEM of three independent experiments. ***P < 0.001 in comparison with control, untreated, cells; NS, non significant.
